# Moderating role of financial ratios in corporate social responsibility disclosure and firm value

**DOI:** 10.1371/journal.pone.0215430

**Published:** 2019-04-18

**Authors:** Muhammad Akram Naseem, Jun Lin, Ramiz ur Rehman, Muhammad Ishfaq Ahmad, Rizwan Ali

**Affiliations:** 1 Lahore Business School, The University of Lahore, Lahore, Pakistan; 2 School of Management, Xi’an Jiaotong University, Xi’an, China; University of Toronto, Rotman School, CANADA

## Abstract

This study investigates the link between corporate social responsibility (CSR) disclosure for multi-stakeholders and financial performance of a firm through accounting-based activities for CSR. A dataset of Chinese non-financial firms listed on the Shanghai Stock Exchange from 2008 to 2012 is taken from the China Stock Market & Accounting Research database. The study compares different financial ratios of CSR disclosure and non-disclosure firms. Moreover, the financial ratios of CSR disclosure firms also compare with the industry averages. The results suggest that the financial of CSR disclosure firms are better than both CSR non-disclosure firms and industry averages. These financial ratios ensure the claim of a firm that they are socially responsible toward multi-stakeholders. Further, the same financial ratios are used as moderator variables between CSR disclosure for multi-stakeholders (independent variable) and firm financial performance (dependent variable). The relationship between CSR disclosure and firm value is moderated by the financial ratios. The moderation effect of financial ratios is rarely used in the literature of CSR disclosure and firm value.

## Introduction

Corporations around the globe are involved in multi-stakeholder corporate social responsibility (CSR) [1] activities. Some firms disclose these activities in annual reports, whereas others publish them on their websites as a separate CSR report. These kinds of activities require expert human resources and involve massive cash outflows. However, the additional information provided to multi-stakeholders may offset the cost of CSR activities. [2] argue that additional information can bridge the gap between stakeholders and a firm and, as a result, affect financial performance. Thus, observing the reaction of stakeholders of a firm that disclose CSR information is essential. [3] argue that a firm’s actions regarding CSR disclosure depend on the expectations of its stakeholders. Creditors and shareholders are significant parties interested in CSR disclosure. Therefore, investigating how multi-stakeholders react to CSR-based expenses incurred by a firm and the rewards of financial payoffs is worthwhile.

The literature of the last 50 years on CSR and firm performance has varied so much that researchers have yet to arrive at any consensus about the linkage between CSR and financial performance [4]. Thus, this debate seems to be far from over. Accordingly, the field of CSR has exponentially grown in the last decade. Three different types of conclusions have been reached by empirical researchers on the association between CSR and financial performance. A negative relationship has been found by some researchers [5, 6]. The likely reasons for such negative relationship are corporate illegalities, antitrust suits, or product problems. Some have observed an inconclusive relationship [7, 8], whereas others have found a positive relationship [9–13]. [14] argue that ambiguity about the relationship between CSR actions and firm performance is due to methodological concerns, model misspecifications, and, most importantly, lack of exploration of the channel through which CSR affects firm value.

This study investigates the role of financial ratios of a firm as a moderator between CSR disclosure for multi-stakeholders and firm financial performance in Chinese context. Little attention has been paid in the literature on this subject to the context of emerging economies as compared with developed markets [15–17]. The study examines different financial ratios such as the proportion of salaries to total expense, advertisement intensity ratio, account payable turnover ratio turnover, interest coverage ratio, and dividend payout moderates the link between CSR disclosure of the respective stakeholders and firm financial performance. To test this relationship, a dataset of Chinese non-financial firms listed on the Shanghai Stock Exchange from 2008 to 2012 is obtained from the China Stock Market & Accounting Research (CSMAR) database [18, 19].

The study establishes unique findings from the perspective of multi-stakeholders; that is, the business’s objective to maximize its value cannot be achieved by ignoring stakeholders. [20] argues that “anyone who can potentially benefit from its engagement with the firm is a stakeholder.” The study contributes to the existing literature of CSR and financial performance that different financial ratios, such as proportion of salaries to total expense, advertisement intensity, account payable turn over, interest coverage ratio and dividend payout moderate the relationship between CSR on disclosure for employee, customer, supplier, creditor and shareholder protection and the firm’s financial performance in China’s non-financial sector.

The rest of this paper is organized as follows. The second section discusses CSR in China and develops hypotheses from the literature review. The third section describes the dataset, variable construction, and models. The fourth section reports the empirical findings and resulting discussion. The last section concludes the paper and offers managerial implications, suggestions, and directions for future research.

## Literature review and theoretical background

Nowadays, the green economy agenda can be timely managed, and shareholders’ goal can be achieved by constantly satisfying the needs of stakeholders [21]. Because of the varying interest of different stakeholders, CSR always remains a matter of discussion between accounting-based and performance-based financial measures. Stakeholders are classified in terms of their different interests of social and ethical responses from any public corporation: 1. shareholders, 2. internal stakeholders (employees), 3. operational stakeholders (customers and suppliers), and 4. society (state authorities, non-governmental organizations, and civil society) [22].

The present study aims to examine the relevance of CSR disclosure to stakeholders and its impact on the financial performance of the firm. The study utilizes agency theory and the idea of information asymmetry to develop testable hypotheses. When a separation of duty between the principal (shareholders) and agents (managers) is upheld, information asymmetry exists [23]. Shareholders need information about the risk exposure of the firm concerning social, environmental, or governance operations and how it affects the firm’s future cash flows and liabilities [24]. Moreover, shareholders want to gain information about management policies to counter risks [25]. The information about the additional risk exposure of the firms affects investment risk assessment and influences firm share prices [26–28]. The worst-case scenario arises when investors do not know relevant information about the company and make adverse share valuation. Therefore, the company with less information disclosure gets a low price of share from investors and pays a high rate of return.

Information asymmetry can be reduced through extensive and relevant disclosure (CSR disclosure in this case) made by the company to their shareholders [28]. CSR disclosure reduces information asymmetry and makes the firm credible and transparent, which result in the reduction of shareholder’s risk assessment [26, 28]. Managers receive incentives with share-based payments. In addition, managers can be removed if share prices are declined at the same time. Accordingly, managers often maximize their incentives by pursuing activities that increase the share price. CSR disclosure, which reduces information asymmetry, is one of the initiatives taken by managers in this regard. At the same time, when a company spends extra cash for image building in the eyes of their internal and operational stakeholders, firm operating inefficiency is reduced [29]

Norms and standards are still in progress. In the current business decade, shareholder values are created through devoted resources into CSR-related activities. To engage in business activities in China, firms have to abide by social morals and business ethics standards set by the Company Law of the People’s Republic of China (2006). However, state-owned companies are required to follow CSR guidelines issued by the Chinese Central Government in 2008. Guidelines for CSR disclosure were issued by the country’s two stock exchanges, Shenzhen and Shanghai, in 2006 and 2008, respectively. These guidelines aim to encourage the incorporation of CSR disclosure in companies’ annual reports. [30] suggests that China has introduced several initiatives about CSR awareness in its corporate sector, but the pressure on Chinese companies from stakeholders to provide more CSR information is still weak. Moreover, corporate information disclosure adds market value of the firms and assesses risk for effective decision making [28].

CSR information is considered weak in China for numerous reasons. An institutional regulatory framework by the Chinese government that can address the cost of irresponsible behaviors of Chinese companies is unavailable. Moreover, consumer awareness and consumer rights are deficient. NGOs are also handicapped by the Chinese government and political environment. The insignificant growth of socially responsible investments in Chinese capital markets compared with their Western counterparts and the lack of community pressure on Chinese firms that exhibit irresponsible behavior toward the environment are the reasons for the weak CSR information in China. [31] note that CSR can be a potential source of competitive advantage and innovation, much more than just a cost, constraint, and charitable deeds.

Socially responsible firms can add potential benefits that can increase a firm value, such as improved labor relations and employee productivity, few complaints from the society, improved brand recognition, low regulatory costs, and environmentally friendly behaviors. Such potential benefits suggest that socially responsible firms can perform better than their counterparts, therefore suggesting a positive association between CSR and the firm [32–36]. This study describes the moderating effect of accounting-based measures with CSR disclosure on market-based measures that explain their combined effect on the firm’s financial performance. Accounting-based measure represents firms’ different accounting procedures and management priorities during financial periods while fulfilling the needs of shareholders. Therefore, managing firm growth while meeting all stakeholder needs is always needed.

### Customer protection disclosure and financial performance

As one of the external stakeholder groups, customers are vitally important irrespective of the type of business. Customers are the reason for the long-term existence of any firm. The CSR for customers is often disclosed by firms in its annual reports as customer protection disclosure. Some studies have examined the general effect of social, environmental, governance, or any related corporate responsibility performance or disclosure on the financial performance of the firms. However, a cross-sectional and longitudinal study conducted in the UK did not confirm any direct association between CSR disclosure and financial performance of a firm [37].

Moreover, [38] find a weak and negative relationship between CSR disclosure and firm financial performance for Australian listed firms. [39] find no relationship between CSR disclosure and firm share price for European listed firms. However, very few studies that examine the relationship between customer protection disclosure and firm financial performance are available in the literature. In the existing literature, the association between CSR and financial performance in the customer channel is examined using structured questionnaires [40] and interviews [41]. [41] argue that customers support firms that are involved in CSR activities. However, customers are also mostly unaware of firms’ CSR activities.

The literature suggests that a proportion of customers are willing to pay high prices for products/services of firms that are involved in CSR [42–44]. However, Penn Schoen Berland [45] suggests that even price-sensitive customers are willing to buy from socially responsible firms. This finding indicates that customer-based CSR helps enhance customer loyalty [46]. [47, 48] argue that CSR-related advertisements and media exposure may increase customer awareness of CSR. In turn, the demand for products/services of CSR-related firms increases, which ultimately improves the financial performance of the firm. [49] find that non-financial disclosures are directly associated with financial performance and customer and investor satisfaction.

These mixed results suggest that evidence in the literature on the direct relationship between CSR disclosure and firm financial performance is weak. Therefore, the current study hypothesizes that tangible actions taken by firms to assure CSR toward customer in terms of higher advertisement intensity ratio moderate the relationship between CSR disclosure for customer protection and firm performance.

*Hypothesis 1: Advertisement Intensity moderates the link between customer protection disclosure and firm financial performance*.

Marketing campaigns are now equipped with pro-environmental and pro-social messages for realizing to their customers that they are building an eco-friendly relationship [50]. Information gap between the company and its users can be removed through advertising intensity, which is one of the most valuable relationship elements. Customer confidence can be potentially enhanced by providing them with more clear information through advertising. Either of this information is embedded with a product or corporate philanthropic activities [51]. Even in the wake of the catastrophic 2008 Sichuan earthquake, corporate philanthropic giving was strategic. Previous research results state that a positive association between advertising intensity and philanthropic behavior of the firm exists [48, 52].

Moreover, [53] prove that advertising intensity plays a moderating role between CSR and marketing performance of the firm. This study uses the ratio of a firm’s advertising to total expenditures as advertisement intensity to assure the customer based CSR disclosure by a firm [54]. It also establishes the moderating effect on the relationship between customer protection disclosure and financial performance, an idea established by [55].

### Employees’ protection disclosure and financial performance

A substantial amount of studies with a wide range of theoretical viewpoints is available. These studies suggest that CSR disclosure, either environmental or social (employees or society), has significance in a corporation performance. However, epistemological differences in the literature are also observed. The results vary across countries, periods, and corporations [56–57]. Studies have proven the relationship between CSR disclosure and firm performance, but the relationship remains inconclusive. [57–59] suggest that CSR disclosure and firm performance have no relationship.

Moreover, [60, 61] find no relationship or negative relationship. However, Bowman [62] finds a positive relationship between CSR social disclosure and financial performance. In modern business practices, the employees of any organization are considered to be the most valuable assets and play an integral role in the achievement of organizational goals. As charity begins at home, employers should first think about the welfare of their employees and fulfill their basic needs. Pro-social organizational behaviors can be built if employees feel that their employers are trustworthy and fair.

In the social psychology field, signaling and social identity theories explain how CSR affects the attitudes and behaviors of firm employees. [63] argue that employees evaluate the firm’s image and reputation by commenting on various organizational signals. CSR activities are considered one of the sources of these signals [64]. When CSR actions are interpreted as reliable indicators of a firm’s values, employees’ perceptions are favorably influenced, and firms can acquire an advantage from such actions [65, 66]. [67] emphasize the importance of alignment between the identities of employees and the organization, and a firm’s investment in its employees strengthens such alignment. In many cases, to retain valuable employees and maintain high moral standards, organizations adopt high labor standards, develop CSR activities, and contribute to the society [68]. Employee-based CSR is one source of employees’ great commitments [69] and a direct association between employees’ well-being and a firm’s CSR activities [69].

In other words, CSR is one of the significant reputational factors that make an organization attractive to current and potential employees [70]. Furthermore, employees who view their organizations as ethical and trustworthy are less likely to quit, thus reducing search, negotiation, and other transaction costs associated with hiring and training new employees [38]. The literature suggests that employees working in firms with low levels of CSR have a low self-image, low job satisfaction, and low productivity when compared with those working in firms with a high level of CSR. That is, the organization’s level of CSR and employees’ self-image, job satisfaction, and productivity are positively associated [71–73].

An organization that is responsive toward philanthropic activities must be more generous toward its employees. If a firm is fulfilling the basic psychological needs (salary) of its employees, then this firm meaningfully follows the CSR practices [74]. Social identical and signaling theories prove that employees’ behaviors can be mold toward CSR activities and company efforts, for instance, by paying average salaries. The literature on employee–organization relationship suggests that salary is one of the critical factors that determine an employee’s job satisfaction, motivation, stress, and productivity [75, 76]. [77] suggests that salary-based CSR of organizations enhances employee participation and job satisfaction, which has a direct relationship with an employee–company identification [78].

In light of the above-mentioned theoretical and empirical evidence, the study hypothesizes that firms that have reasonable pay structure concerning their total expense (financial ratio) moderate the relationship between CSR employee protection disclosure and firm performance.

*Hypothesis 2: Proportion of salaries to total expense moderates the link between employee protection disclosure and financial performance of the firm*.

### Supplier protection disclosure and firm financial performance

In today’s business world, organizations understand the significance of selecting appropriate suppliers who can maintain their supply chain promptly without compromising quality[79, 80]. Krause, [81] note that an excellent relationship with a supplier can create many opportunities for organizations [82, 83]. Ideas, capabilities, and timely supply of quality material are some benefits of supplier–organization relationships. These benefits create competitive advantages that may not be otherwise obtained [84]. However, [85] and [86] explain that competing firms may search for similar benefits from their supplier base. Social exchange theory indicates that for supplier satisfaction, exchanges are not confined to material goods but are also based on intangibles [87].

*Hypothesis 3: Account payable turnover moderates the link between supplier protection disclosure and financial performance of the firm*.

[88] describe satisfaction as perceived emotions of fairness when the results are obtained in a buyer-supplier relationship. However, [89] demonstrate through a mixed methods research that the behavior and business environment are the most important factors in a buyer-supplier relationship and supplier satisfaction. Quality relationships with suppliers may create active cooperation. CSR for suppliers can be viewed as (1) valuing the independence of suppliers, (2) fair agreements with suppliers, and (3) performing based on the agreement.

The corporate strategies of organizations can be measured based on their CSR practices [90]. CSR may sometimes increase the costs of products or interrupt the cash flows of an organization. However, cash outflows, including prompt creditor payments, may also build a foundation for organizations to strengthen their relationships with their stakeholders. To achieve sustainable development, the consequences above should be affordable. Quick payments to suppliers create additional relational capital, and a positive corporate reputation can attract more investors. Each industry has different types of stakeholders. For instance, compared with other types of stakeholders, suppliers are given more importance in the manufacturing industry and play indispensable roles in services or non-manufacturing industries. Quality norms are always appreciated regardless of the nature of an industry. Accounts payable turnover (APTO) ratio is a short-term accounting liquidity measure that reflects how quickly an organization pays its liabilities to its suppliers. A reduction in APTO indicates that an organization is not making quick payments to its suppliers, which can affect the buyer-supplier relationship and the satisfaction of suppliers as well as signify the worsening financial performance of the firm. Numerous studies have established a positive link between buyer-supplier relationship and financial performance [91, 92] that can lead to superior responsiveness and customer loyalty [92], innovation, and quality [91, 93].

### Creditor protection disclosure and financial performance

Financial stability is always a key focus area in business strategies. [94] classified stakeholders into the three main classes of legitimacy, power, and urgency based on their relationships and classified creditors into the two influential classes of legitimacy and power. According to stakeholder theory, as external stakeholders, creditors play a pivotal role in achieving the goals of an organization. When the organization functions well, its shareholders manage the firm as its owners. Meanwhile, when the organization faces a financial crisis or insolvency, creditors can officially take over the firm and exercise their control. In this way, creditors are also essential stakeholders of organizations. The CSR of creditors can be seen in their (1) timely payment of debts and (2) lawful implementation of contracts.

*Hypothesis 4: Interest coverage ratio moderates the link between creditor protection disclosure and the financial performance of the firm*.

[95] argued that creditors might influence the CSR and financial performance of organizations. In testing such argument, [96] found a negative relationship between the debt ratios and CSR of organizations, while [97] found a positive relationship between creditors and level of CSR disclosure.

Creditors sometimes do not participate in decision-making processes but instead focus on the liquidity ratio of their organizations. A sound interest earned ratio strengthens the performance of a firm and boosts its social and ethical practices. Those organizations that show poor welfare initiatives toward their creditors are considered high risk. Apart from the nature of their industries, external pressure can drive organizations toward implementing different strategies to satisfy the needs of their stakeholders [90]. This study uses the interest coverage ratio as a measure for the ability of organizations to pay interest and assure the social responsibility of these organizations toward their creditors. Interest coverage ratio may also indicate the profitability and solvency of an organization. To maintain a normal level of solvency, the interest coverage ratio must be at least greater than 1. A higher interest coverage ratio indicates higher solvency.

### Shareholder protection disclosure, and financial performance

Managers of organizations make decisions on how they can handle the conflicting demands of their stakeholders while simultaneously achieving their strategic goals. Managers are always under pressure due to the conflicting demands of their stakeholders. Stakeholder theory posits that decision makers must pay equal attention to the demands of their shareholders and other stakeholders (e.g., creditors, employees, suppliers, customers, and the society at large) to fulfill their expectations. Occasionally, other vital stakeholders are overvalued compared with other shareholders, and such imbalance affects the financial performance of an organization [94, 98].

*Hypothesis 5: Dividend per share moderates the link between shareholder protection disclosure and the financial performance of the firm*.

[99] found that each investor responds to CSR differently; specifically, individual investors are less sensitive to the CSR initiatives of an organization compared with institutional investors. Furthermore, the positive effects of CSR on the responses of an investor depend on the CSR disclosure level of the organization.

The long-term benefits of CSR investment are time-dependent. With regard to effective stakeholder relationships, [65] argued that only those firms with a real dedication to CSR are likely to obtain long-term benefits from such investment; otherwise CSR investment leads to cost. Responsibility to shareholders is considered a primary responsibility of organizations. Companies pay dividends to their shareholders to maintain their interest and to attract additional investors. These organizations also spend much effort in establishing and maintaining positive relationships with their shareholders to facilitate the achievement of their goals. The CSR initiatives of organizations for their shareholders can be reflected in their (1) timely disclosure of information, (2) protection of shareholders’ rights, (3) allocation of profit to shareholders, and (4) commitment to improving both the earnings of the firm and the profits of the shareholders.

[100] highlighted the importance of CSR disclosure in reducing the information gap between managers and investors as well as other stakeholders. Given that CSR disclosure can be used as a tool for the supervision and control of managers, having an efficient board of directors can emphasize and promote CSR disclosure. Previous studies reveal that CSR disclosure may positively influence the awareness of stakeholders about the performance, value, risk, profitability, cost of capital, and share price of an organization [101–104].

Managers want to do some social good for their stakeholders even if doing so is beyond the interests of their organizations. To promote their CSR, managers sometimes respond in favor of other stakeholders while completely ignoring the needs (especially for profit maximization) of their most important stakeholders. Continuity in dividend payments is considered a positive indicator not only for the shareholders but also for other stakeholders. CSR policy is positively associated with a greater dividend payout ratio [105]. This study uses dividends per share to assure CSR of organizations toward their shareholders under the assumption that the distribution of dividends informs the shareholders about the financial conditions of an organization. Investor confidence is a determinant of dividend distribution, which also affects the confidence of other stakeholders and ensures the stability of an organization.

## Data and variable construction

### Sample

The sample includes all Chinese firms listed on the Shanghai Stock Exchange from 2008 to 2012. The selected sample period is vital for CSR disclosure because, in 2008, the Shanghai Stock Exchange issued some regulations to encourage all listed Chinese companies to disclose CSR information in their financial reports. The financial data of non-financial listed Chinese companies are collected from the China Stock Market and Accounting Research (CSMAR). A total of 1500 companies from non-financial industries are included in the sample [18, 106]. The firm-level financial and CSR disclosure data of Chinese listed firms are collected from the CSMAR database and the annual reports of these organizations.

### Descriptive statistics

Table 1 presents the descriptive statistics of the entire sample, including the minimum, maximum, mean, and standard deviation. The average values of EPS and Tobin’s q are positive at 0.36 and 2.11, respectively. The total assets (TA) of the selected organizations vary from 0 to 1247 billion RMB, while their average total assets are equal to 4.21 billion RMB.

**Table 1:** Descriptive statistics: Full sample.

	Min	Max	Mean	Std. Dev
PS	0.00	0.42	0.04	0.06
APTO	0.00	55.23	1.17	3.71
DPS	-3.54	22.43	0.04	0.31
ICR	-45243.05	11261.47	1.41	541.34
AI	0.00	0.19	0.02	0.06
EPS	-21.86	12.82	0.36	0.62
Tobin’ s q	-7.67	389.22	2.11	6.04
Total Assets(TA)*	0.00	1,247	4.21	28.61

*: Billion RMB (Amount Ren Min Bi (RMB) Chinese currency)

### Model specifications

The moderating effect of CSR for multi-stakeholders on the relationship between CSR disclosure and the financial performance of organizations is determined by using the following models.

#### Model 1: Advertisement intensity as a moderator

$$Tobin\prime sq_{it}=\alpha_{it}+\beta_{1}\text{log}TA_{it}+\beta_{2}AI_{it}+\beta_{3}CUSP_{it}+\beta_{4}CUSP_{it}*AI_{it}+\varepsilon_{it}$$ (1)

Model 1 describes the moderating effect of CSR for customers on customer protection disclosure (CUSP) and firm financial performance. Tobin’s q is used as a proxy for the market value of a firm [64, 107], whereas the advertising intensity (AI) is measured as the financial ratio of advertising expenditures to the total operating expenditures in a given financial year. CUSP is a dummy variable that takes a value of 1 if the firm discloses customer protection information in its financial statements; otherwise, this variable takes a value of 0. The log of total assets (log TA) is used as a control variable[47, 107].

#### Model: 2 Proportion of salaries to total expense as a moderator

$$Tobin\prime sq_{it}=\alpha_{it}+\beta_{1}\text{log}TA_{it}+\beta_{2}PS_{it}+\beta_{3}STP_{it}+\beta_{4}PS_{it}*STP_{it}+\varepsilon_{it}$$ (2)

In model 2, PS is measured as the ratio of employees’ wages to the total operating expenditures of an organization in a given financial year. Employee’s protection disclosure (STP) is a dummy variable that takes a value of 1if the firm discloses employee protection information in its financial statements; otherwise, this variable takes a value of 0.

#### Model 3: APTO as a moderator

$$Tobin\prime sq_{it}=\alpha_{it}+\beta_{1}\text{log}TA_{it}+\beta_{2}APTO_{it}+\beta_{3}DP_{it}+\beta_{4}APTO_{it}*DP_{it}+\varepsilon_{it}$$ (3)

In Model 3, APTO is an account payable turnover ratio. Supplier protection disclosure (DP) is a dummy variable that takes a value 1 if the firm discloses supplier protection information in its financial statements; otherwise, this variable takes a value of 0.

#### Model 4: Interest coverage ratio as a moderator

$$Tobin\prime sq_{it}=\alpha_{it}+\beta_{1}\text{log}TA_{it}+\beta_{2}ICR_{it}+\beta_{3}CP_{it}+\beta_{4}ICSR_{it}*CP_{it}+\varepsilon_{it}$$ (4)

Model 4 uses an interest coverage ratio (ICR) to assure CSR disclosure for creditor protection. Creditor protection disclosure (CP) is a dummy variable that takes a value of 1 if the firm discloses creditor protection information in its financial statements; otherwise, this variable takes a value of 0.

#### Model 5: Dividend per share as a moderator

$$Tobin\prime s\;q_{it}=\alpha_{it}+\beta_{1}\text{log}TA_{it}+\beta_{2}DPS_{it}+\beta_{4}SP_{it}+\beta_{4}DPS_{it}*SP_{it}+\varepsilon_{it}$$ (5)

Model 5 explains the moderating role of dividend per share on the relationship between shareholder protection information disclosure and firm financial performance. Shareholder protection disclosure (SP) is a dummy variable that takes a value of 1 if the firm discloses shareholder protection information in its financial statements; otherwise, this variable takes a value of 0. Table 2 summarizes the variables used in this work and how they are operationalized.

**Table 2:** Variable construction.

SN	Name	Abbreviation	Definition
1	Tobin’s q	Tobin’s q	The market value of the firm divided by total assets
2	Total assets	TA	Sum of all assets presented on the balance sheet
3	Advertising intensity	AI	The ratio of advertising expense to total expenditure
4	Proportion of salary	PS	The ratio of salary expense to total expenditure
5	Accounts payable turnover	APTO	The ratio of total purchases to average accounts payable
6	Interest coverage ratio	ICR	The ratio of a company’s earnings before interest and taxes to total interest expense
7	Dividends per share	DPS	The ratio of total dividends paid out to the number of shares.
8	Shareholder protection	SP	Binary variable, ‘1’ if the firm is involved in and discloses its shareholder protection activities, otherwise ‘0.’
9	Creditor protection	CP	Binary variable, ‘1’ if the firm is involved in and discloses its creditor protection activities, otherwise ‘0.’
10	Staff protection	STP	Binary variable, ‘1’ if the firm is involved in and discloses its staff protection activities, otherwise ‘0.’
11	Delivery protection	DP	Binary variable, ‘1’ if the firm is involved in and discloses its delivery protection activities, otherwise ‘0.’
12	Customer Protection	CUP	Binary variable, ‘1’ if the firm is involved in and discloses its customer protection activities, otherwise ‘0.’

Each of the above models represents the residual term and is assumed to follow all assumptions of the classic linear regression model. To estimate these models and to avoid outliers, Tobin’s q is winsorized at the 1st and 99th percentiles. The missing values of the other variables are treated as equal to 0 [108, 109]. Panel data regression analysis is performed to determine the dynamics of change between CSR disclosure and financial performance because panel data provide “more information, more variability, less collinearity among variables, more degrees of freedom and more efficiency” [110]. The models are then estimated by employing pooled regression, fixed effects, and random effects via the ordinary least squares method (OLS). Standard errors are clustered to address the lack of independence in the observations [111]. All standard errors are adjusted to address heteroscedasticity issues [112]. [112] test is performed in choosing between the fixed effects and random effects models, while Wald test is performed in choosing between the fixed effects and pooled regression models. Given that both of these tests reject the possibility of using random effects or pooled regression models, using a fixed effects model is considered appropriate. Therefore, only the results of the fixed effects regression model are reported in this paper.

## Empirical findings

### CSR to multi-stakeholder’s accounting measure

This study uses different financial ratios to assure the CSR information disclosure for various stakeholder by Chinese firms. Comparison of the means test is performed to check for any significant differences in the financial ratios of those firms that disclose and do not disclose their CSR for their respective stakeholders.

Table 3 shows that the average dividends per share (DPS) significantly differ between those firms that disclose and do not disclose their shareholder protection information. This finding indicates that when disclosing its shareholder protection information in its financial reports, an organization ensures its disclosure by paying a higher dividend. These results establish a high dividend payout ratio assure the claim of a firm CSR disclosure for the shareholder. Meanwhile, those firms that disclose creditor protection information have a statistically significant and high ICR. Having a high ICR improves the performance of organizations and drives them to engage in social and ethical practices. This result lends empirical support that the ICR ensure the creditor protection disclosure of a firm. Those firms that disclose their employee protection information show a statistically significant and high PS ratio. APTO is an important accounting ratio for firms to maintain sustainable relationships with their suppliers. Table 3 shows that those firms that disclose their supplier protection information have an average APTO of 1.79, which is statistically significant and higher than that of firms that do not disclose such information. This finding indicates that those firms which claim that they are protecting the rights of their suppliers are more concerned and efficient in making and clearing inventory payments compared with other firms. Those firms that disclose their customer protection information have an average AI ratio of 1.75, which is higher and statistically significant compared with that of firms that do not disclose such information. Previous studies provide sufficient evidence to support the use of AI as an indicator of customer based CSR [48, 52, 53], and the findings of this work lend further empirical support that by using AI one can assure the CSR customer protection disclosure of a firm.

**Table 3:** Comparison of CSR item disclosures.

Proxies	Disclosure Item	Status	Average	Z-stat	Sig.
Dividends per share (DPS)	Shareholder Protection	Disclosed	1.87	-6.857	0.00
		Non-Disclosed	1.65		
Interest coverage ratio (ICR)	Creditor Protection	Disclosed	1.75	-1.939	0.05
		Non-Disclosed	1.66		
Proportion of salaries (PS)	Employee Protection	Disclosed	1.83	-5.814	0.00
		Non-Disclosed	1.65		
Accounts payable turnover (APTO)	Delivery Protection	Disclosed	1.79	-3.952	0.00
		Non-Disclosed	1.66		
Advertisement intensity (AI)	Customer Protection	Disclosed	1.75	-5.297	0.00
		Non-Disclosed	1.66		

Table 4 presents the comparison of the means test for financial ratios of industry and firms which disclose CSR for multi-stakeholders. The results indicate that the average financial ratios of CSR disclosed firms are statistically significant and higher than the respective industry averages. For example, the average proportion of salaries ratio is significantly higher for those firms which disclose employee protection disclosure as compared to the industry average.

**Table 4:** Average financial ratio comparison between industry and CSR disclosures firms.

SN	Industry	PS			APTO			DPS			ICR			AI		
		Ind Avg	DCSR-Avg	t-stat/ (Sig)	Ind Avg	DCSR-Avg	t-stat/ (Sig)	Ind Avg	DCSR-Avg	t-stat/ (Sig)	Ind Avg	DCSR-Avg	t-stat/ (Sig)	Ind Avg	DCSR-Avg	t-stat/ (Sig)
1	Agriculture, forestry, animal husbandry, fishery	1.02	1.92	2.78*** (0.00)	0.87	1.68	2.93*** (0.00)	1.01	1.35	2.67* (0.05)	1.07	1.27	2.91** (0.01)	1.15	1.61	2.36* (0.08)
2	Extractive Industries, Electricity, gas and water production and supply industry	1.03	1.85	2.59** (0.01)	0.99	1.79	2.75** (0.01)	1.02	1.53	2.57* (0.06)	1.09	1.71	3.58*** (0.00)	1.23	1.52	2.72** (0.04)
3	Manufacturing, Wholesale and retail trade	1.08	1.83	3.92*** (0.00)	1.12	1.77	2.26** (0.04)	1.03	1.99	3.10*** (0.00)	1.12	1.79	3.49*** (0.00)	1.17	1.82	2.95** (0.01)
4	Building industry, Real Estate	1.05	1.92	2.95*** (0.00)	1.14	1.78	2.97*** (0.00)	1.01	1.55	3.15*** (0.00)	1.15	1.53	2.73* (0.05)	1.12	1.49	2.83** (0.03)
5	Transportation, warehousing, IT industry, Social Services, Communication and cultural industries	1.07	1.83	2.48* (0.05)	1.19	1.79	2.85*** (0.00)	1.03	1.77	1.89* (0.07)	0.11	1.79	2.61* (0.06)	1.01	1.69	2.85** (0.01)

Note:

*, ** and *** indicate significance at the 10%,5%, and 1% levels, respectively

### Regression results

Table 5 presents the results of the panel data regression models with fixed effects that are used to test hypotheses 1 to 5 and to examine the moderating effect of financial ratios to explain the link between CSR disclosure and financial performance as perceived by different stakeholders. The estimated results of models (1) to (5) are also presented in this table. The positive and significant coefficients of the CSR disclosure items in these models all suggest that the disclosure of CSR activities improves the financial performance of organizations. (Model 1) highlights the importance of AI for companies given that all of its coefficients, including that of CUSP and the interaction term (AI×CUSP), are found to be statistically significant. In other words, AI plays an important role in improving the financial performance of an organization and shows a moderating effect on the relationship between customer protection disclosure and firm performance.

**Table 5:** Panel regression -Tobin’s q.

Items	Model-1	Model-2	Model-3	Model-4	Model-5
	Customers	Employees	Suppliers	Creditors	Shareholders
Intercept	1.69(0.00)***	1.53(0.00)***	-1.32(0.00)***	0.35(0.00)***	0.34 (0.00)***
Log assets	0.05 (0.03)**	0.59(0.01)**	0.03(0.00)***	0.07(0.00)***	0.04(0.00)***
Salary		0.75(0.00)***			
AI	0.33(0.00)***				
APTO			-0.13(0.00)***		
ICR				0.24(0.00)***	
DPS					0.24(0.00)***
SP (Shareholder protection)					0.39(0.00)***
CP (Credit Protection)				0.07(0.08)*	
DP (Delivery Protection)			0.15(0.00)***		
CusP (Customer Protection)	0.69(0.00)***				
StP (Staff Protection)		0.17(0.00)***			
SALARY×StP		0.53(0.03)**			
APTO×DP			0.03(0.06)*		
ICR×CP				0.04(0.00)***	
AI×CusP	0.67(0.05)**				
DPS×SP					0.23(0.00)***
Year Dummies	YES	YES	YES	YES	YES
Industries Dummies	YES	YES	YES	YES	YES
Firm fixed effects	YES	YES	YES	YES	YES
Adjusted R^2^	0.47	0.45	0.49	0.44	0.47
N	7555	7555	7555	7555	7555
Hausman Test /Chi-Square	29.57(0.00)***	31.21(0.00)***	17.52(0.01)***	79.13(0.00)***	69.90(0.00)***
Wald Test /Chi-Square	135.43(0.00)***	189.46(0.00)***	59.51(0.00)***	134.53(0.00)***	115.46(0.00)***

Note:

*, ** and *** indicate significance at the 10%,5%, and 1% levels, respectively

(Model 2) considers the perspectives of employees. The coefficient of salaries paid to employees ratio and its interaction with employee protection disclosure are both statistically significant, thereby suggesting that firms can improve their financial performance by disclosing their activities related to employee protection.

Meanwhile, the coefficient of APTO and its interaction with DP are statistically significant and describe the moderating effect of APTO on the relationship between delivery protection disclosure and financial performance. (Model 3) considers the perspective of suppliers, and the results lend support to hypothesis 3.

(Model 4) considers the perspective of creditors and uses ICR, which regression coefficient and interaction with CP are statistically significant, thereby highlighting the moderating effect of ICR on the relationship between creditor protection disclosure and firm performance. Similarly, given that shareholders are important groups of stakeholders, DPS moderates the relationship between SP and financial performance. Similarly, in (Model 5), the coefficient of DPS and its interaction with shareholder protection disclosure are both significant.

## Discussion and conclusion

By highlighting the causal relation between CSR disclosure and several variables, this study confirms that a high level of CSR disclosure captures the attention of stakeholders and that CSR disclosure may be used to resolve agency issues. This study empirically tests the relationship between CSR activities and financial performance of Chinese listed firms while considering the importance of different types of stakeholders. The results suggest that CSR disclosure can improve the performance of organizations as manifested in the significant differences between the financial performances of those companies that disclose and do not disclose their CSR activities.

A firm that discloses information about its CSR for multi-stakeholders is highly valued by investors in the market when certain financial ratios assure the disclosure information. This study contributes to the existing literature of CSR disclosure in several ways. Firstly, the use of financial ratios ensures the firm’s CSR disclosure claim for multi-stakeholders. For instance, if a firm discloses employee’s protection disclosure, then its proportion of salaries ratio should be better than those firms which do not claim such disclosure. Secondly, the study uses financial ratios as a moderator to explain the link between CSR disclosure for multi-stakeholders and financial performance of a firm. The results of the study suggest that certain financial ratios make the assurance of a firm’s CSR disclosure. Such as, the APTO ratio is better and significantly higher for those firm which discloses supplier protection disclosure as compared to those firms which do not. Moreover, the average APTO of firms which disclosed CSR information is higher and significant as compared to the industry average.

Further, the results of this work are intuitive and empirically justify the selection of financial ratios as a moderator between CSR disclosure and firm performance. Specifically, with a high-interest coverage ratio, those firms that disclose their creditor protection information outperform those firms that do not disclose such information. When accounting and financial aspects are not considered, one cannot easily determine how CSR disclosure increases the value of a firm. Therefore, this study sheds light on how the multi-stakeholder CSR disclosure of firms can increase their value in the presence of better financial ratios. For example, if these firms claim that they protect the rights of their suppliers and make timely payments for its inventories, then they can achieve a positive partnership with their suppliers and subsequently increase their value. These results echo the findings from the literature. For instance, [20] proposed enlightened stakeholder theory to resolve the fundamental conflict between the neo-classical approach of [113], which posits that managers focus on maximizing the wealth of shareholders at the expense of ignoring their other stakeholders, and the stakeholder theory of [60], which posits that managers must show equal responsibility to all their stakeholders. The findings of this work support the argument of [20], who claimed that managers could increase the wealth of their shareholders without necessarily ignoring the welfare of their other stakeholders. However, China has a distinct organizational culture and environment compared with western countries. Chinese companies have a limited yet steadily growing awareness of CSR and its practices compared with other firms from developed markets.

Our work proposes some directions for future research. For instance, how executives and other firms that do not engage in CSR perceive the concept of CSR and CSR disclosure must be explored to establish a link between corporate governance and CSR communication. Some interesting questions can also be addressed in exploring such a link. Does poor corporate governance drive the negative association between CSR activities and financial performance? How does a change in governance affect CSR activities? Addressing these questions and the link between CSR activities and the agency problem is a fruitful avenue for further research in this field. Furthermore, a mixed methodology may also be adopted in future investigations to shed novel findings on this topic.

## Supporting information

S1 Dataset The data for empirical analysis. (RAR)

## References

[1] Pedram A, Pedram P, Yusoff NB, Sorooshian S. Development of closed–loop supply chain network in terms of corporate social responsibility. PlOS ONE. 2017; 12(4): e0174951 10.1371/journal.pone.0174951 28384250 PMC5383151

[2] Cormier D, Ledoux MJ, Magnan M. The informational contribution of social and environmental disclosures for investors. Management Decision. 2011; 49(8): 1276–1304.

[3] Nielsen AE, Thomsen C. Reporting CSR–what and how to say it? Corporate Communications: An International Journal. 2007; 12(1): 25–40.

[4] De Bakker FG, Groenewegen P, Den Hond F. A bibliometric analysis of 30 years of research and theory on corporate social responsibility and corporate social performance. Business & Society. 2005; 44(3): 283–317.

[5] Marcus A. The deterrent to dubious corporate behavior: Profitability, probability and safety recalls. Strategic Management Journal. 1989; 10(3): 233–250..

[6] Vance S. C. (1975). Are socially responsible corporations good investment risks. Management Rreview. 1975; 64(8): 19–24.

[7] Aupperle KE, Carroll AB, Hatfield JD. An empirical examination of the relationship between corporate social responsibility and profitability. Academy of Management Journal. 1985; 28(2): 446–463.

[8] Dahlsrud A. How corporate social responsibility is defined: an analysis of 37 definitions. Corporate Social Responsibility and Environmental Management. 2008;15(1):1–13..

[9] Belkaoui A. The impact of the disclosure of the environmental effects of organizational behavior on the market. Financial Management, 1976; 26–31.

[10] Hart S, Ahuja G. Does it Pay to be Green? Paper presentado en la National meetings of the Academy of Management, Dallas. Texas. 1994.

[11] Johnson RA, Greening DW. Relationships between corporate social performance, financial performance, and firm governance. Paper presented at the Academy of Management Proceedings. 1994..

[12] Wang X, Kevin Huang S, Yang CL. Corporate social performance: why it matters? Case of Taiwan. Chinese Management Studies. 2014; 8(4): 704–716.

[13] Riahi-Belkaoui A. Executive compensation, organizational effectiveness, social performance and firm performance: An empirical investigation. Journal of Business Finance & Accounting. 1992; 19(1): 25–38.

[14] Margolis JD, Walsh JP. People and profits? The search for a link between a company’s social and financial performance: Psychology Press. 2001.

[15] Gray R, Bebbington J. Corporate sustainability, accountability and the pursuit of the impossible dream. Handbook of Sustainable Development. 2007; 376–394.

[16] Smith JVDL, Adhikari A, Tondkar RH. Exploring differences in social disclosures internationally: A stakeholder perspective. Journal of Accounting and Public Policy. 2015; 24(2): 123–151.

[17] Williams SM, Pei CAHW. Corporate social disclosures by listed companies on their web sites: An international comparison. The International Journal of Accounting. 1999; 34(3): 389–419.

[18] Haniffa RM, Cooke TE. The impact of culture and governance on corporate social reporting. Journal of Accounting and Public Policy. 2005;24(5): 391–430.

[19] Li Q, Luo W, Wang Y, Wu L. Firm performance, corporate ownership, and corporate social responsibility disclosure in China. Business Ethics: A European Review. 2013;22(2):159–173.

[20] Jensen M. Value maximisation, stakeholder theory, and the corporate objective function. European Financial Management. 2001; 7(3): 297–317.

[21] Karagiorgos T. Corporate social responsibility and financial performance: An empirical analysis on Greek companies. European Research Studies. 2010; 13(4): 85.

[22] Cummings L, Patel C. Managerial attitudes toward a stakeholder prominence within a Southeast Asia context: Emerald Group Publishing Limited. 2009.

[23] Deegan C. Organizational legitimacy as a motive for susutainability reporting. Sustainability Accounting and Accountability. 2007: 127–149.

[24] Al-Tuwaijri SA, Christensen TE, Hughes II.The relations among enviornmnetal disclosure, enviornmental performance, and economic performance: a simultabeous equations approcah. Accounting, Organizations and Society.2004;29,5(6): 447–471.

[25] Clarkson PM, Li Y, Richardson GD, Vasvari FP. Revisiting the relation between enviornmental performance and enviornmental disclosure: An emprical analysis. Accounting, Organizations and Society.2008; 33,4(5): 303–327.

[26] Cormier D, Magnan M, Van Velthoven B. Envionmental disclosure quality in large German companies:Economic incentives, public pressures or institutional conditions? European Accounting Review. 2005; 14(1): 3–39.

[27] Dhaliwal DS, Li OZ, Tsang A, Yang YG. voluntary nonfinancial disclosure and the cost ofequity capital: The Initiation of Corporate Social Responsibility Reporting. The Accounting Review 2011; 86(1): 59–100.

[28] Healy P M, Palepu K G. Information asymmetry, corporate disclosure, and the capital markets: A review of the empirical disclosure literature. Journal of Accounting and Economics. 2001; 31(1–3): 405–440.

[29] Yang FJ, Lin CW, Chang YN. The linkage between corporate social performance and corporate financial performance. African Journal of Business Management. 2010; 4(4): 406–413.

[30] Lin LW. Corporate social responsibility in China: Window dressing or structural change. Berkeley Journal of International Law. 2010; 28(1): 64.

[31] Carroll AB, Shabana KM. The business case for corporate social responsibility: A review of concepts, research and practice. International Journal of Management Reviews. 2010; 12(1): 85–105.

[32] De Klerk M, De Villiers C. The value relevance of corporate responsibility reporting: South African evidence. Meditari Accountancy Research. 2012; 20(1): 21–38.

[33] Villiers CD, Staden CJV. Shareholders’ requirements for corporate environmental disclosures: A cross country comparison. The British Accounting Review. 2010; 42(4): 227–240.

[34] Goll I, Rasheed AA. The moderating effect of environmental munificence and dynamism on the relationship between discretionary social responsibility and firm performance. Journal of Business Ethics. 2004; 49(1): 41–54.

[35] Marom IY. Toward a unified theory of the CSP–CFP link. Journal of Business Ethics. 2006; 67(2): 191–200.

[36] Ruf BM, Muralidhar K, Brown RM, Janney JJ, Paul K. An empirical investigation of the relationship between change in corporate social performance and financial performance: A stakeholder theory perspective. Journal of Business Ethics. 2001; 32(2): 143–156.

[37] Murray SO, Boyaci H, Kersten D. The representation of perceived angular size in human primary visual cortex. Nature Neuroscience. 2005; 9(3): 429–434. 10.1038/nn1641 16462737

[38] Jones TM. Instrumental stakeholder theory: A synthesis of ethics and economics. Academy of Management Review. 1995; 20(2): 404–437.

[39] Reverte C. The impact of better corporate social responsibility disclosure on the cost of equity capital. Corporate Social Responsibility and Enviornmental Management. 2012, 19(5): 253–72.

[40] Chomvilailuk R, Butcher K. The effect of CSR knowledge on customer liking, across cultures. International Journal of Bank Marketing. 2013; 31(2): 98–114.

[41] Green T, Peloza J. How do consumers infer corporate social responsibility? The role of organisation size. Journal of Consumer Behaviour. 2014; 13(4): 282–293.

[42] Bhattacharya CB, Sen S. Doing better at doing good: When, why, and how consumers respond to corporate social initiatives. California Management Review. 2004; 47(1): 9–24.

[43] Bosch-Badia MT, Montllor-Serrats J, Tarrazon-Rodon MA. Corporate social responsibility: A real options approach to the challenge of financial sustainability. PlOS ONE. 2015; 10(5), e0125972 10.1371/journal.pone.0125972 25938410 PMC4418608

[44] Sen S, Bhattacharya CB. Does doing good always lead to doing better? Consumer reactions to corporate social responsibility. Journal of Marketing Research. 2001; 38(2): 225–243.

[45] Penn Schoen Berland. Corporate Social Responsibility Branding Survey. 2010. http://www.slideshare.net/BMGlobalNews/csr-branding-survey-2010-final.

[46] Wei YC, Egri CP, Lin YY. Do corporate social responsibility practices yield different business benefits in eastern and western contexts? Chinese Management Studies. 2014; 8(4): 556–576.

[47] McWilliams A. Corporate social responsibility. Wiley Encyclopedia of Management. 2000.

[48] McWilliams A, Siegel D. Corporate social responsibility: A theory of the firm perspective. Academy of Management Review. 2001; 26(1): 117–127.

[49] Maines LA, Bartov E, Fairfield P M, Hirst D E, Iannaconi T E, Mallett R, et al Recommendations on disclosure of nonfinancial performance measures. Accounting Horizons. 2004; 16(4): 353–362.

[50] Yoon S, Kim Y, Baek TH. Effort investment in persuasiveness: a comparative study of environmental advertising in the United States and Korea. International Journal of Advertising. 2016; 35(1): 93–105.

[51] Dick AS, Basu K. Customer loyalty: toward an integrated conceptual framework. Journal of the Academy of Marketing Science. 1994; 22(2): 99–113.

[52] Zhang R, Zhu J, Yue H, Zhu C. Corporate philanthropic giving, advertising intensity, and industry competition level. Journal of Business Ethics. 2010; 94(1): 39–52.

[53] Rahman M, Rodríguez-Serrano MÁ, Lambkin M. Corporate Social Responsibility And Marketing Performance: The Moderating Role Of Advertising Intensity. Journal of Advertising Research. 2017; 57(4): 368–378.

[54] Servaes H, Tamayo A. The impact of corporate social responsibility on firm value: The role of customer awareness. Management Science. 2013; 59(5): 1045–1061.

[55] Bagwell K. The economic analysis of advertising. Handbook of Industrial Organization.2007; 3: 1701–1844.

[56] Ullmann AA. Data in search of a theory: A critical examination of the relationships among social performance, social disclosure, and economic performance of US firms. Academy of Management Review. 1985; 10(3): 540–557.

[57] Fry FL, Hock RJ. Who claims corporate responsibility? The biggest and the worst. Business and Society Review. 1976; 18(18): 62–65.

[58] Mobeva JM, Ortas E. Are stock markets influenced by susainability matter? Evidence from European companies. International Journal of Sustainable Economy. 2008; 1(1): 1–16.

[59] Hackston D, Milne MJ. Some determinants of social and environmental disclosures in New Zealand companies. Accounting, Auditing & Accountability Journal. 1996; 9(1): 77–108.

[60] Freeman RE. Strategic management: A stakeholder approach. Boston: Pitman 1984.

[61] Freedman M, Jaggi B. An analysis of the association between pollution disclosure and economic performance. Accounting, Auditing & Accountability Journal. 1988; 1(2): 43–581.

[62] Bowman MJ, Okubo A. Cabbeling at thermohaline fronts. Journal of Geophysica research.1978; 83: 6173–6178.

[63] Fombrun C, Shanley M. What’s in a name? Reputation building and corporate strategy. Academy of Management Journal. 1990; 33(2): 233–258.

[64] Waddock SA, Graves SB. The corporate social performance-financial performance link. Strategic management journal. 1997; 303–319.

[65] Barnett ML. Stakeholder influence capacity and the variability of financial returns to corporate social responsibility. Academy of Management Review. 2007; 32(3): 794–816.

[66] Barnett ML, Salomon RM. Does it pay to be really good? Addressing the shape of the relationship between social and financial performance. Strategic Management Journal. 2012; 33(11): 1304–1320.

[67] Collier J, Esteban R. Corporate social responsibility and employee commitment. Business Ethics: A European Review. 2007; 16(1): 19–33.

[68] Hartman LP, Rubin RS, Dhanda KK. The communication of corporate social responsibility: United States and European Union multinational corporations. Journal of Business Ethics. 2007; 74(4): 373–389.

[69] Yu Y, Choi Y. Corporate social responsibility and firm performance through the mediating effect of organizational trust in Chinese firms. Chinese Management Studies. 2014; 8(4): 577–592.

[70] Lindgreen A, Swaen V, Johnston WJ. Corporate social responsibility: An empirical investigation of US organizations. Journal of Business Ethics. 2009; 85(2): 303–323.

[71] Albinger HS, & Freeman SJ. Corporate social performance and attractiveness as an employer to different job seeking populations. Journal of Business Ethics. 2000; 28(3): 243–253.

[72] Backhaus KB, Stone BA, Heiner K. Exploringthe relationship between corporate social performance and employer attractiveness. Business & Society. 2002; 41(3): 292–318.

[73] Greening DW, Turban DB. Corporate social performance as a competitive advantage in attracting a quality workforce. Business & Society. 2000; 39(3): 254–280.

[74] Rupp DE, Ganapathi J, Aguilera RV, Williams CA. Employee reactions to corporate social responsibility: An organizational justice framework. Journal of Organizational Behavior: The International Journal of Industrial, Occupational and Organizational Psychology and Behavior. 2006; 27(4): 537–543.

[75] Judge TA, Cable DM, Higgins CA. The employment interview: A review of recent research and recommendations for future research. Human Resource Management Review. 2000; 10(4): 383–406.

[76] Oshagbemi T. Correlates of pay satisfaction in higher education. International Journal of Educational Management. 2000; 14(1): 31–39.

[77] Wagner JA. Participation’s effects on performance and satisfaction: A reconsideration of research evidence. Academy of Management Review. 1994; 19(2): 312–330.

[78] Kim HR, Lee M, Lee HT, Kim NM. Corporate social responsibility and employee–company identification. Journal of Business Ethics. 2010; 95(4): 557–569.

[79] Michaels RE, Kumar A, Samu S. Activity-specific role stress in purchasing. Journal of Supply Chain Management. 1995; 31(1): 11.

[80] Lii WEP. Use of human judgment models in industrial buyers’ vendor selection decisions. Industrial Marketing Management. 1996; 25(2): 135–149.

[81] Krause DR, Handfield RB, Tyler BB. The relationships between supplier development, commitment, social capital accumulation and performance improvement. Journal of Operations Management, 2007; 25(2): 528–545.

[82] Bernardes ES, Zsidisin GA. An examination of strategic supply management benefits and performance implications. Journal of Purchasing and Supply Management. 2008; 14(4): 209–219.

[83] Nyaga GN, Whipple JM, Lynch DF. Examining supply chain relationships: do buyer and supplier perspectives on collaborative relationships differ? Journal of Operations Management. 2010; 28(2): 101–114.

[84] Koufteros X, Vickery SK, Dröge C. The effects of strategic supplier selection on buyer competitive performance in matched domains: does supplier integration mediate the relationships? Journal of Supply Chain Management. 2012; 48(2): 93–115.

[85] Takeishi A. Knowledge partitioning in the interfirm division of labor: The case of automotive product development. Organization Science. 2002; 13(3): 321–338.

[86] Dyer JH, Hatch NW. Relation-specific capabilities and barriers to knowledge transfers: creating advantage through network relationships. Strategic Management Journal. 2006; 27(8): 701–719.

[87] Homans GC. Social behavior as exchange. American Journal of Sociology. 1958; 63(6): 597–606.

[88] Essig M, Amann M. Supplier satisfaction: Conceptual basics and explorative findings. Journal of Purchasing and Supply Management. 2009; 15(2): 103–113.

[89] Hüttinger L, Schiele H, Schröer D. Exploring the antecedents of preferential customer treatment by suppliers: a mixed methods approach. Supply Chain Management: An International Journal. 2014; 19(5/6): 697–721.

[90] Chen L, Feldmann A, Tang O. The relationship between disclosures of corporate social performance and financial performance: Evidences from GRI reports in manufacturing industry. International Journal of Production Economics. 2015; 170: 445–456.

[91] Johnston DA, McCutcheon DM, Stuart FI, Kerwood H. Effects of supplier trust on performance of cooperative supplier relationships. Journal of Operations Management. 2004; 22(1): 23–38.

[92] Martin JH, Grbac B. Using supply chain management to leverage a firm’s market orientation. Industrial Marketing Management. 2003; 32(1): 25–38.

[93] Corsten D, Felde J. Exploring the performance effects of key-supplier collaboration: an empirical investigation into Swiss buyer-supplier relationships. International Journal of Physical Distribution & Logistics Management. 2005; 35(6): 445–461.

[94] Mitchell RK, Agle BR, Wood DJ. Toward a theory of stakeholder identification and salience: Defining the principle of who and what really counts. Academy of Management Review. 1997; 22(4): 853–886.

[95] Roberts RW. Determinants of corporate social responsibility disclosure: An application of stakeholder theory. Accounting, organizations and society. 1992; 17(6): 595–612.

[96] Branco MC, Rodrigues LL. Factors influencing social responsibility disclosure by Portuguese companies. Journal of Business Ethics. 2008; 83(4): 685–701.

[97] Huang CL, Kung FH. Drivers of environmental disclosure and stakeholder expectation: Evidence from Taiwan. Journal of Business Ethics. 2010; 96(3): 435–451

[98] Donaldson T, Preston LE. The stakeholder theory of the corporation: Concepts, evidence, and implications. Academy of Management Review. 1995; 20(1): 65–91.

[99] Sheikh NA, Wang Z. Determinants of capital structure: An empirical study of firms in manufacturing industry of Pakistan. Managerial Finance. 2011; 37(2): 117–133.

[100] Jamali D, Safieddine AM, Rabbath M. Corporate governance and corporate social responsibility synergies and interrelationships. Corporate Governance: An International Review. 2008; 16(5): 443–459.

[101] Ghoul SE, Guedhami O, Kwok CC, Mishra DR. Does corporate social responsibility affect the cost of capital? Journal of Banking & Finance. 2011; 35(9): 2388–2406.

[102] Lourenço IC, Branco M, Curto JD, Eugénio T. How does the market value corporate sustainability performance? Journal of Business Ethics. 2012; 108(4): 417–428.

[103] Scholtens B. Corporate social responsibility in the international banking industry. Journal of Business Ethics. 2009; 86(2): 159–175.

[104] Simpson WG, Kohers T. The link between corporate social and financial performance: Evidence from the banking industry. Journal of Business Ethics. 2002; 35(2): 97–109.

[105] Semenescu A, Curmei CV. Using CSR to mitigate information asymmetry in the banking sector. Management & Marketing. 2015; 10(4): 316–329.

[106] Li Q, Luo W, Wang Y, Wu L. Firm performance, corporate ownership, and corporate social responsibility disclosure in China. Business Ethics: A European Review. 2013; 22(2): 159–173.

[107] Morck R, Shleifer A, Vishny RW. Management ownership and market valuation: An empirical analysis. Journal of financial economics. 1988; 20: 293–315.

[108] Fee CE, Hadlock CJ, Pierce JR. Investment, financing constraints, and internal capital markets: Evidence from the advertising expenditures of multinational firms. The Review of Financial Studies. 2008; 22(6): 2361–2392.

[109] Hale G, Santos JA. Do banks price their informational monopoly? Journal of Financial Economics. 2009; 93(2): 185–206.

[110] Gujarati D. Econometrics by example: Palgrave Macmillan. 2014.

[111] White H. A heteroskedasticity-consistent covariance matrix estimator and a direct test for heteroskedasticity. Econometrica: Journal of the Econometric Society. 1980; 48(4): 817–838.

[112] Hausman J. Specification tests in econometrics. Econometrica. 1978; 46(6): 1251–1271.

[113] Friedman M. The social responsibility of business is to make profits. New York Review. 1970; 13.

